# Bigelovin triggered apoptosis in colorectal cancer *in vitro* and *in vivo* via upregulating death receptor 5 and reactive oxidative species

**DOI:** 10.1038/srep42176

**Published:** 2017-02-09

**Authors:** Mingyue Li, Li-Hua Song, Grace Gar-Lee Yue, Julia Kin-Ming Lee, Li-Mei Zhao, Lin Li, Xunian Zhou, Stephen Kwok-Wing Tsui, Simon Siu-Man Ng, Kwok-Pui Fung, Ning-Hua Tan, Clara Bik-San Lau

**Affiliations:** 1 School of Biomedical Sciences, The Chinese University of Hong Kong, Shatin New Territories, Hong Kong; 2 Institute of Chinese Medicine, The Chinese University of Hong Kong, Shatin New Territories, Hong Kong; 3 School of Traditional Chinese Medicines, China Pharmaceutical University, Nanjing 211198, China; 4 State Key Laboratory of Phytochemistry and Plant Resources in West China (CUHK), The Chinese University of Hong Kong, Shatin New Territories, Hong Kong; 5 State Key Laboratory of Phytochemistry and Plant Resources in West China, Kunming Institute of Botany, Chinese Academy of Sciences, Kunming 650201, China; 6 Department of Surgery, The Chinese University of Hong Kong, Shatin, New Territories, Hong Kong

## Abstract

Colorectal cancer (CRC) is the third most prevalent cancer and the third highest cancer-related mortality in the United States. Bigelovin, a sesquiterpene lactone isolated from *Inula helianthus aquatica*, has been proven to induce apoptosis and exhibit anti-inflammatory and anti-angiogenic activities. However, the effects of bigelovin on CRC and underlying mechanisms have not been explored. The present study demonstrated that bigelovin exhibited potent anti-tumor activities against CRC *in vitro* and *in vivo*. Bigelovin suppressed cell proliferation and colony formation and induced apoptosis in human colorectal cancer HT-29 and HCT 116 cells *in vitro*. Results also revealed that bigelovin activated caspases, caused the G2/M cell cycle arrest and induced DNA damage through up-regulation of death receptor (DR) 5 and increase of ROS. In HCT 116 xenograft model, bigelovin treatment resulted in suppression of tumor growth. Bigelovin at 20 mg/kg showed more significant tumor suppression and less side effects than conventional FOLFOX (containing folinic acid, 5-fluorouracil and oxaliplatin) treatment. In addition, *in vivo* data confirmed that anti-tumor activity of bigelovin in CRC was through induction of apoptosis by up-regulating DR5 and increasing ROS. In conclusion, these results strongly suggested that bigelovin has potential to be developed as therapeutic agent for CRC patients.

Colorectal cancer is one of the top three most common cancer and the third leading cause of cancer-related death in the United States^1^. In 2016, American Cancer Society (ACS) estimates that 134,490 persons will be diagnosed having colorectal cancer (CRC), and more than one-third will die from this cancer^2^. Although the incidence and mortality rate of CRC in developed countries declined during the last decade mainly due to the early screen in asymptomatic and average risk people^2^,^3^, incidence and mortality rate are still growing in developing world with increasing westernized lifestyle and aging population^3^. Surgery is the primary treatment for most of the CRC patients^4^. For patients in higher level of metastatic stage, radiation and chemotherapy often accompany with surgery. Currently, fluorouracil (5-Fu) is often used alone or combined with folinic acid and oxaliplatin as FOLFOX to treat primary colon cancer. For advanced or metastatic CRC, FOLFOX and FOLFIRI (5-Fu, folinic acid and irinotecan) are the most commonly used chemotherapy combinations. Despite the effectiveness of the chemotherapy and radioactive therapy, the high incidence (up to 98%) of side effects, including hair loss, nausea, vomiting, neurotoxicity, increasing the chance of infection and immune system suppression often affect the quality of life^5^,^6^. Targeted therapy to vascular endothelial growth factor (e.g. bevacizumab) or epidermal growth factor receptor (e.g. cetuximab) are common adjuvant/alternative treatments for CRC^7^. Although they are reported to increase survival rates for cancer patients, the costs for these treatments are high^8^. Hence, the searching of new compounds from natural source with high efficacy, low toxicity and low cost for CRC remains highly desirable.

Traditional Chinese medicines (TCM) have been used for thousands of years for treating various diseases, however, the active components and mechanisms are still often unanswered. In the past, natural compounds have been proven as rich sources of anticancer drugs, such as paclitaxel, camptothecin^9^,^10^. Bigelovin, a sesquiterpene lactone isolated from *Inula helianthus-aquatica*, was identified as a selective retinoid X receptor α agonist^11^, possessed anti-emetic^12^ activities, anti-angiogenic activities^13^, and down-regulated the gene expressions of inflammatory-related cell adhesion molecules and monocyte adhesion^14^. Previous studies demonstrated that bigelovin could induce apoptosis on a panel of cancer cell lines including leukemia, lung, liver, glioma, kidney, gastric, cervix and breast *in vitro*^15^,^16^. However, the anti-CRC effect and the underlying mechanisms of bigelovin have not been investigated.

Death receptor 5 (DR5) is a TNF-related apoptosis inducing ligand (TRAIL) receptor, which has been identified as a novel target with better selectivity for cancer therapy as shown to induce apoptosis in a diversity of cell types^17^. Engagement of DR 5 results in the activation of caspase 8, which in turn activates downstream effector caspases in the extrinsic apoptosis pathway. While reactive oxygen species (ROS) are known to be regulator of TRAIL receptor induction^18^. Furthermore, emerging evidences illustrated that other terpenoids such as eriocalyxin B^19^, celastrol^20^, tagalsins^21^ and zerumbone^22^ can cause ROS-mediated apoptosis due to their structure of α, β-unsaturated ketone moieties^19^. Bigelovin also has two α, β-unsaturated ketone moieties (Fig. 1a), thus we hypothesized that bigelovin-induced apoptosis may be mediated by ROS.

The present study aimed to investigate the inhibitory effect of bigelovin on CRC through evaluating its anti-tumor effect *in vivo* and elucidating the underlying mechanisms of actions *in vitro*.

## Results

### Bigelovin inhibited growth and colony formation of human colon cells

**Figure 1:**
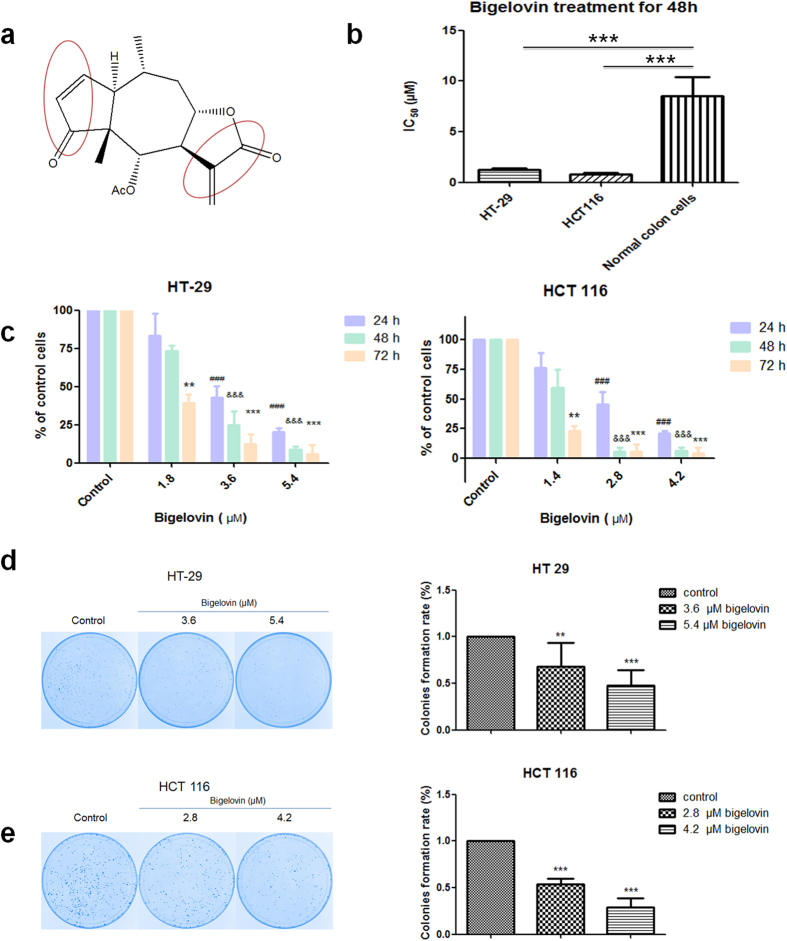
Bigelovin inhibited cell viability and proliferation in human colon cancer cells. (**a**) Chemical structure of bigelovin with two α, β-unsaturated ketone moieties. (**b**) Bigelovin was selectively toxic to colorectal cancer cells comparing to primary normal colon cells. IC_50_ values from 48 h incubation in HT-29 and HCT 116 cell lines and primary normal colon cells by MTT assay. (Mean ± SD; ***p < 0.001, vs primary normal colon cells; n = 4). (**c**) Cell proliferation assay of two cell lines treated with bigelovin for indicated dose and time points (**p < 0.01, ***^,###,&&&^p < 0.001 vs. medium control at the corresponding time point; n = 3–4). HT-29 (**d**) and HCT 116 (**e**) were seeded in 100 mm dish, and after 48 h bigelovin treatment, they were reseeded and maintained for 8 or 11 days to form colonies (**p < 0.01, ***p < 0.001 vs. medium control; n = 4–5).

Cell viability was assessed by MTT assay on HT-29 and HCT 116 cells. Cells were treated with bigelovin (0.037 to 9 μM) or 5-Fu/cisplatin (0.11 to 27 μM) for 24, 48 and 72 h. As shown in the Supplementary Table S1, colon cancer cell lines were more sensitive to bigelovin treatment rather than 5-Fu or cisplatin. Bigelovin induced cytotoxicity in these two cancer cell lines in time-dependent and dose-dependent manners. To test the selectivity of bigelovin, primary human colon cells were used (mixture of fibroblast and epithelial cells, data not shown). From IC_50_ values, primary colon cells were less sensitive to bigelovin treatment (8.55 μM for 48 h treatment) comparing to colon cancer cell lines (0.8 and 1.2 μM for 48 h treatment, Fig. 1b and Table S1). To test the effects of bigelovin on cell proliferation, HT-29 and HCT 116 cells were treated with bigelovin at 1.4–5.4 μM (1 to 3 folds of 24 h IC_50_ values for each cell line) for 24, 48 and 72 h. As shown in Fig. 1c, bigelovin significantly reduced cell proliferation of both cell lines in a time- and dose- dependent manners. Further more, to determine cell lethal- and sub-lethal damage repair after bigelovin treatment, HT-29 and HCT 116 cells were reseeded and maintained for 8 or 11 days to allow colony formation. Cells which were treated by bigelovin showed significantly decreased colony formation ability as compared with vehicle control (Fig. 1d,e). The decreasing of colony formation ability indicated that bigelovin could decrease damage repair ability of colon cancer cell lines. Taken together, our results showed that bigelovin suppressed the growth of colorectal cancer cells.

### Bigelovin induced apoptosis through caspases activation

**Figure 2:**
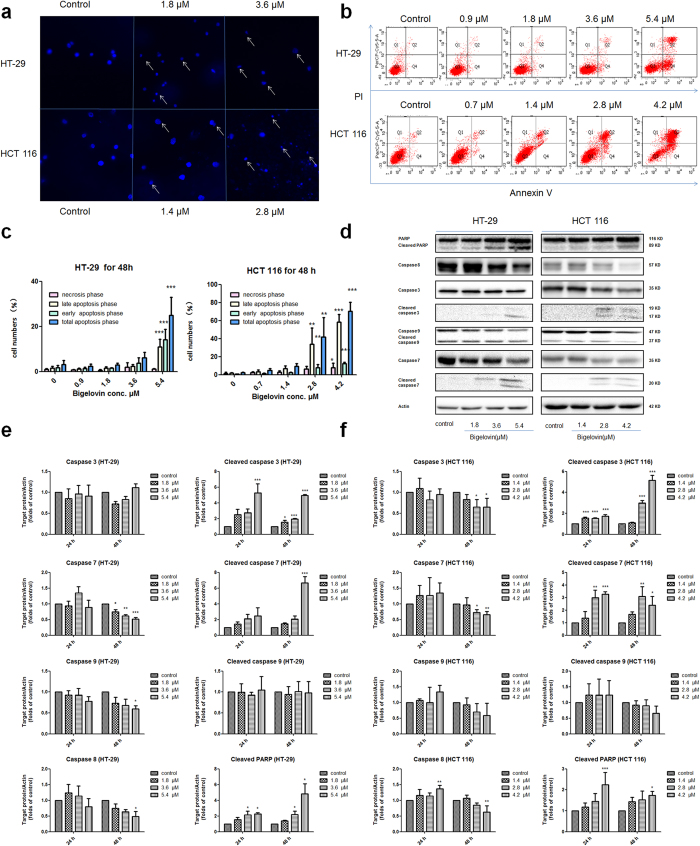
Bigelovin induced apoptosis in colorectal cancer cells. (**a**) Morphological alterations of HT-29 and HCT 116 cells after 48 h bigelovin treatment stained with Hoechst 33258. White arrows indicated the cells exhibiting chromatin condensation or bulge and bleb. (**b**) Bigelovin induced apoptosis in HT-29 and HCT 116 at the indicated doses for 48 h was evaluated by Annexin V and PI double staining and were quantified (**c**). Mean ± SD; *p < 0.05, **p < 0.01, ***p < 0.001 vs. medium control at the same time point; n = 3–4. (**d**) HT-29 and HCT 116 were treated with bigelovin for 48 h at indicated doses, then whole cell extracts were prepared and analyzed by Western blot using antibodies against PARP, caspases 3, 7, 8, 9 and cleaved caspase 3, 7, 9 and the results were quantified (**e** for HT-29, **f** for HCT 116). The results were represented as mean ± SD; *p < 0.05, **p < 0.01, ***p < 0.001 vs. medium control at the corresponding time point (n = 3–4).

Suppression of cancer growth arises from inducing of apoptosis, inhibition of cell proliferation, or both^23^. Bigelovin could inhibit cell proliferation as shown above, hence, whether bigelovin-induced potent effects mediated by apoptosis in two colon cancer cell lines were also examined. Two cancer cells were treated with bigelovin and then stained with Hoechst 33258. After bigelovin treatment for 48 h, morphologies of two cancer cell lines were dramatically altered (Fig. 2a). Most of the bigelovin-exposed cells were shrunken and detached from the substratum of the culture plate. To further confirm whether cell growth inhibition of bigelovin was associated with induction of apoptosis, Annexin V and PI double staining using flow cytometry were used. In this assay, comparing to control, after bigelovin treatment, more cells were undergoing early apoptosis (Q4) and late apoptosis (Q2) ranging from 2% to 70% (Fig. 2b). The apoptotic inducing effects appeared in dose- and time- dependent manners with significant differences compared to control group (Fig. 2c). Next, we examined the effect of bigelovin on the activation of caspases 3, 7, 8, 9, poly (ADP-ribose) polymerase (PARP) and their cleaved forms. Caspase 8 (linking the extrinsic pathway), caspase 9 (linking the intrinsic pathway) are “initiator” caspases, while caspase 3 and caspase 7 are “effector” caspases. PARP-1 is a guardian for genome by functioning in DNA damage surveillance whose cleavage by caspase is regarded as a hallmark of apoptosis and an early marker for chemotherapy-induced apoptosis^24^. The “initiator” and “effector” caspases were all activated and expression of marker protein (cleaved PARP) increased significantly in time- and dose-dependent manners in HT-29 (Fig. 2d,e) and HCT 116 (Fig. 2d,f) after bigelovin treatment. These results indicated that bigelovin could induce apoptosis by caspase activation.

### Bigelovin caused G2/M arrest and DNA damage through regulating Cyclin B1, p-Rb, and p-H2AX

**Figure 3:**
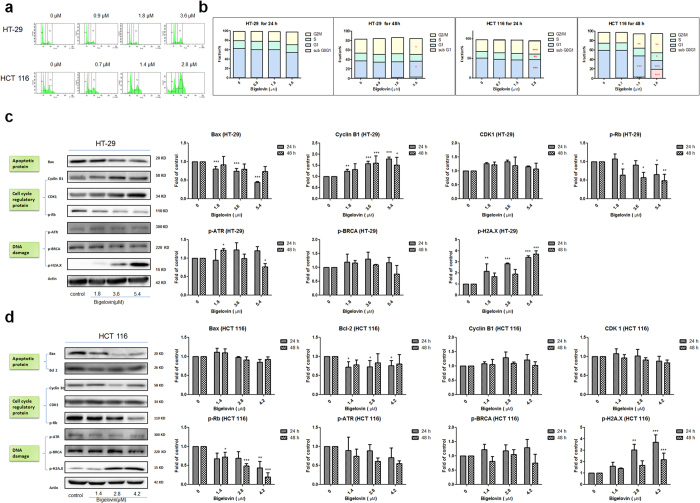
Bigelovin caused G2/M cell cycle arrest and DNA damage. Cell cycle of two cancer cell lines treated with bigelovin (48 h) was evaluated by flow cytometry (**a**), and the results (24 h and 48 h bigelovin treatment) were quantified (**b**). The results were represented as the mean ± SD; *p < 0.05, **p < 0.01, ***p < 0.001 vs. medium control at the same time point. n = 3–4. Western blot analysis of apoptosis proteins, cell cycle regulatory proteins and DNA damage marker proteins in HT-29 (**c**) and HCT 116 (**d**) at indicated doses and quantitative data which were normalized with the protein level of actin and expressed as fold of control. The results were represented as mean ± SD; *p < 0.05, **p < 0.01, ***p < 0.001 vs. medium control at the same time point (n = 3–4).

The commonality of cancers is to proliferate beyond constraints and suppress apoptosis^25^. Cell cycle regulators are the linker between proliferation and apoptosis. Effect of bigelovin on cell cycle of cancer cells was examined. Firstly, two colorectal cancer cells were treated with bigelovin for 24 or 48 h and analyzed by flow cytometry. Bigelovin treatment (3.6 μM for HT-29 and 2.8 μM for HCT 116 cells, Fig. 3a,b) led to a significant increase in the population of cells in G2/M phase at 48 h in both cell lines. From the quantitative data (Fig. 3b), the effects of bigelovin were in dose- and time- dependent manners. As cyclin B1 and CDK 1 are the critical targets for G2/M checkpoint^26^, and play crucial roles in mitotic catastrophe and mitosis, in order to further explore the mechanism of G2/M phase arrest, the expressions of cyclin B1, CDK 1 and p-Rb were analyzed by Western blot. As shown in Fig. 3c (HT-29), expression of cyclin B1 was significantly up-regulated. Similarly, bigelovin-treated HCT 116 cells (Fig. 3d) could be observed in an increasing trend of cyclin B1 expression. Expression of p-Rb decreased significantly in both cells. In addition, DNA damage will cause G_2_ phase delay to provide time for repair before initiation of mitosis^27^. Phosphorylation of histone H2AX at Ser139 (γ-H2AX) is considered as a marker of DNA double strand breaks, which is the most detrimental form of DNA damage^28^. To confirm DNA damage induction, two cell lines were treated with bigelovin and the levels of p-H2AX (a hallmark of DNA damage), as well as other DNA damage-related proteins (p-ATR and p-BRCA) were determined by Western blot. We found that the level of p-H2AX was significantly raised upon bigelovin treatment (Fig. 3c,d). However, the intrinsic pathway regulatory proteins, Bax and Bcl2 were decreased in HT-29 (Fig. 3c) and HCT 116 (Fig. 3d), respectively, indicating that they might not be the key molecules of bigelovin-induced apoptosis.

### Extrinsic pathway plays an important role and DR5 is a target for bigelovin-induced apoptosis

**Figure 4:**
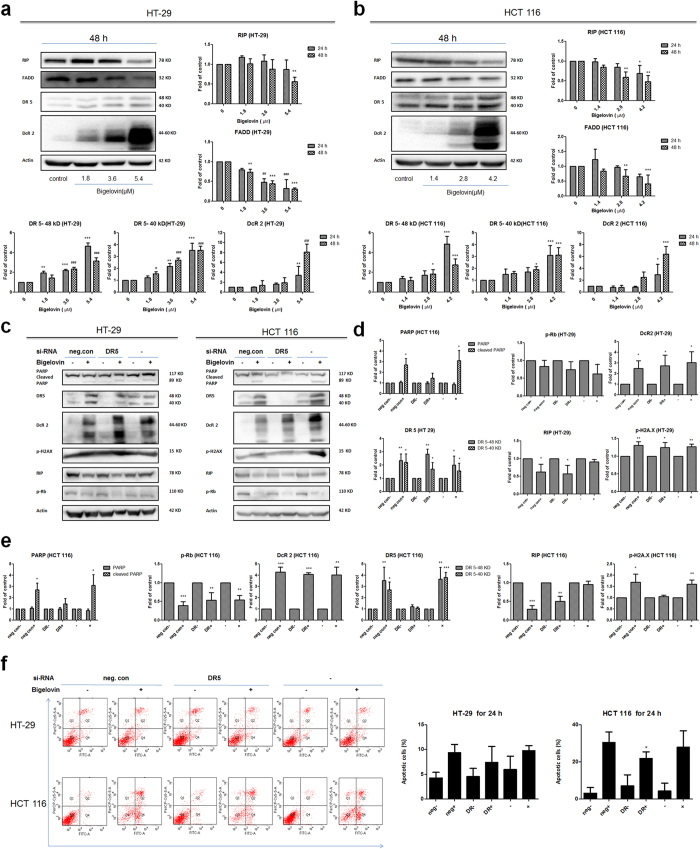
Bigelovin activated extrinsic apoptosis pathway and DR 5 is a target for bigelovin-induced apoptosis. Western blot analysis of protein expression involved in bigelovin-induced extrinsic pathway in HT-29 (**a**) and HCT 116 (**b**) at indicated doses at 48 h and quantitative data, adjusted relative to actin protein level and expressed as fold of control (mean ± SD for 3–4 independent experiments). Two cell lines were transfected with DR 5 siRNA or control siRNA alone. After 24 h, cells were treated with bigelovin (5.4 μM for HT-29 and 4.2 μM for HCT 116 cells) for additional 24 h and subjected to Western blot analysis (**c**–**e**) and Annexin V and PI double staining by flow cytometry (**f**). (**d**,**e**) Are quantitative data for Western blot and (**f**) is quantitative data for Annexin V and PI double staining. Each assay was performed for at least 3 times and presented in mean ± SD; *p < 0.05, **p < 0.01, ***p < 0.001 vs. siRNA transfection control at the same time point.

As the changes of initiator caspase activation (caspase 9 in Fig. 2e,f) and apoptosis regulatory proteins (Bax and Bcl-2 in Fig. 3c,d) in intrinsic pathway were not so obvious,in order to prove whether bigelovin induced apoptosis through extrinsic pathway, a panel of proteins (DR5, DcR2, RIP and FADD) involved in extrinsic pathway were analyzed by Western blot. The increases of death receptor (DR5 and DcR2) expressions were caused by bigelovin treatment in both cell lines HT-29 (Fig. 4a) and HCT 116 (Fig. 4b), accompanying with the decrease in the the total form of receptor-interacting protein (RIP). Since bigelovin increased the levels of DR5 in colorectal cancer cells, the importance of DR5 in bigelovin-induced apoptosis were also examined. To validate the requirement of DR5, DR5 expressions after bigelovin treatment were down-regulated using DR5-specific siRNA (Fig. 4c–e) in both cell lines (24 h) and the expressions of apoptosis marker-cleaved PARP were found to be partly reversed (Fig. 4c–e). At the same time, expression changes of other key molecules such as DcR2, p-Rb and RIP were not significantly reversed by DR5-specific siRNA except p-H2AX in HCT 116 cells. In Annexin V and PI double staining assay, HCT 116 cells, which were transfected with DR5 siRNA showed less apoptosis compared to the cells transfected with control siRNA after bigelovin treatment (Fig. 4f). In summary, bigelovin could up-regulated DR5 expression, and loss of DR5 expression partly reversed bigelovin-mediated apoptosis.

### NAC and Z-VAD-FMK inhibited bigelovin-induced apoptosis

**Figure 5:**
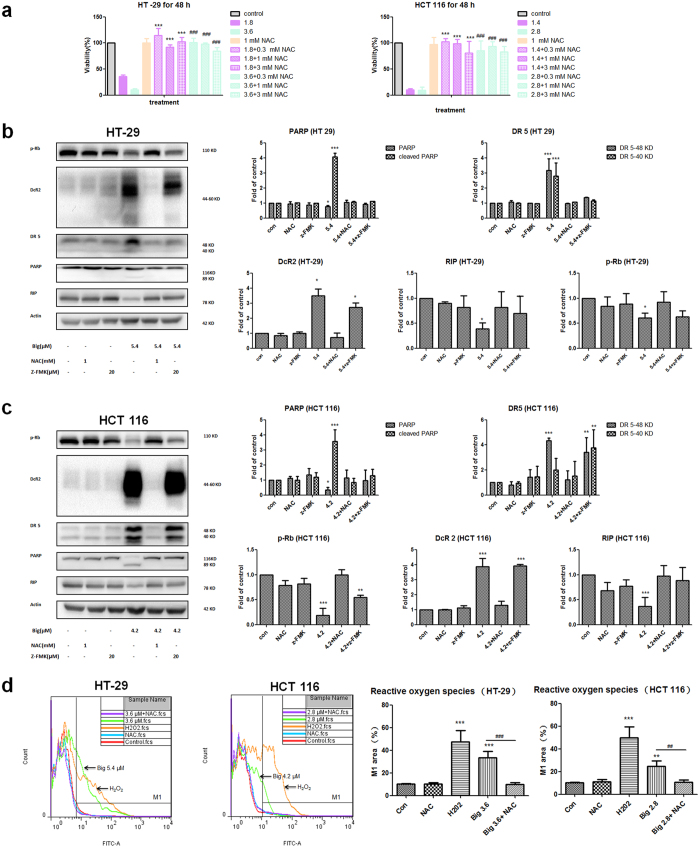
NAC (ROS inhibitor) and Z-VAD-FMK (caspase inhibitor, Z-FMK) could reverse bigelovin-induced apoptosis. (**a**) HT-29 and HCT 116 cells were pretreated with indicated concentrations of NAC for 1 h, then cells were treated with bigelovin for 48 h to detect cell viability by MTT assay. Left panel in (**b**,**c**), cells were pretreated with 1 mM NAC or 20 μM Z-FMK for 1 h, then 24 h bigelovin additional treatment and whole cell proteins were analyzed by Western blot using relevant antibodies. Right panel in (**b**,**c**) are quantitative data for Western blot and data were presented in mean ± SD; *p < 0.05, **p < 0.01, ***p < 0.001 vs. control at the same time point; n = 3–4. Left panel of (**d**), representative histogram showing the relative intracellular ROS of cells stained with CM-H_2_DCFDA dye detected by flow cytometry. Cells were treated with vehicle control (Red line), 1 mM NAC (Blue line), 100 μM H_2_O_2_ (Orange line), 2.8 μM bigelovin (Green line), and 1 mM NAC plus 2.8 μM bigelovin (Purple line). Right panel of (**d**), quantitative effects of bigelovin on ROS production in HT-29 and HCT 116 cell lines. The values are the means ± SD of 3–4 independent experiments; **p < 0.01, ***p < 0.001 vs. vehicle control; ^##^p < 0.01, ^###^p < 0.001 vs. bigelovin treatment at indicated doses (3.6 μM for HT 29 cells and 2.8 μM for HCT 116 cells).

To further verify the role of caspase in bigelovin-induced apoptosis, a pan-caspase inhibitor Z-VAD-FMK (Z-FMK) was used to block caspase activation before bigelovin treatment which can partly inhibit bigelovin-induced apoptosis with regulation of key molecules expression (cleaved PARP and RIP). NAC is ROS scavenger, bigelovin-induced cell death was completely abolished by NAC (Fig. 5a) and the expression of key molecules (DR5, DcR2, PARP, RIP and p-Rb) in bigelovin-induced apoptosis were reversed by NAC pretreatment (Fig. 5b,c). To investigate whether bigelovin could directly affect ROS levels, cells stained with the cell-permeable dye CM-H_2_DCFDA were analyzed by flow cytometry^19^. As shown in Fig. 5d, bigelovin or H_2_O_2_ caused a significant elevation of the ROS level compared with untreated cells in these two cell lines, while ROS levels in cells treated with NAC plus bigelovin treatment returned to the basal level. Thus, combining cytotoxic effects, key molecules expression changes and ROS level alteration, bigelovin-induced apoptosis was linked to bigelovin-induced ROS generation.

### Bigelovin could induce p53 expression

Cyclin-dependent kinase inhibitor p21 is a negative regulator of the cell cycle, so p21 expression was examined. Data showed that bigelovin increased p21 expression in time-dependent manner with maximum at 12 h in both cells (Fig. S1a,b). It indicated that bigelovin regulated cell cycle through regulating p21. In addtion, p21 is up-regulated by the p53 tumor suppressor gene and DR5 is a p53-responsive, downstream gene of the p53^29^, so expression of p53 was also examined. As shown in Fig. S1b, in p53 wild type HCT 116 cells, bigelovin increased p53 expression in time-dependent manner with maximum at 12 h. At 12 h after bigelovin treatment, p53 expression reached maximum at more than 3 folds compared to 0 h. In contrast, in p53 mutant type HT-29 cells, after bigelovin treatment, p53 level did not change significantly (Fig. S1a). Thus, p53 may not play roles in the induction of DR5 by bigelovin.

### Bigelovin inhibited the growth of HCT 116-tumor xenografts in nude mice

**Figure 6:**
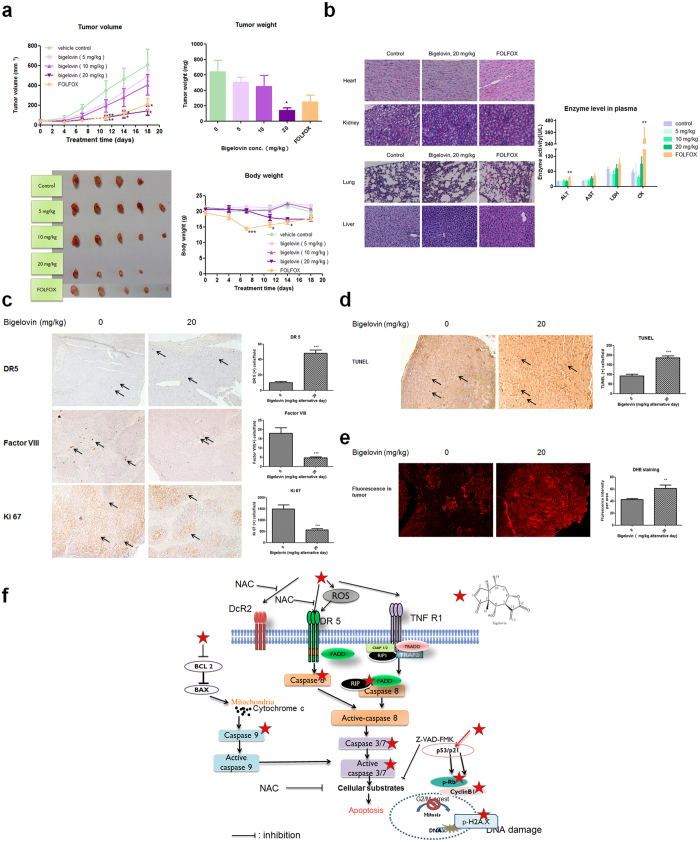
Bigelovin suppressed tumor growth of HCT 116-derived tumor xenografts in nude mice model. HCT 116 cells were subcutaneously injected into the back of nude mice. Mice were treated with vehicle control, bigelovin or FOLFOX. Mean ± SEM; *p < 0.05, **p < 0.01, ***p < 0.001 vs. control at the same time point. (**a**) Tumor growth curve and body weight were calculated twice a week, tumor weight was measured at the end of the experiment. Representative data for 4–5 tumors in each group. (**b**) Organs were examined by H&E staining and plasma enzyme activities of ALT, AST, LDH and CK were calculated at the end of the experiment. (**c**) Tumor tissues were examined by IHC staining with antibodies against DR5, factor VIII and Ki67. (**d**) TUNEL analysis of bigelovin effects on tumor tissues in control and 20 mg/kg bigelovin treatment group. Representative images of 4–5 samples from each group in paraffin-embedded tissue sections. (**e**) Detection of superoxide levels by DHE staining in cryosections from tumors. The mean fluorescent intensity was obtained from 2–3 random visual fields of each tumor and quantified by ImageJ software. (**f**) The putative working model of bigelovin against colorectal cancer: bigelovin induces ROS and increases DR5 expression, then activate downstream caspase and cause G2/M and DNA damage through regulating p21, p-Rb and p-H2AX expression.

As shown in Fig. 6a, for the 5 and 10 mg/kg treatment group, there was a trend of decrease of tumor size, although there was no significant difference between treated and untreated control group. However, bigelovin at 20 mg/kg significantly reduced the tumor volume and final tumor weight. The results illustrated that there was a dose- dependent and time-dependent effect of bigelovin on tumor volume. It is noteworthy that although FOLFOX was effective in reducing tumor size, its severe side effects on body weight were significantly decreased after 7 days of treatment. Photos of tumors (Fig. 6a) clearly showed the dose-dependent effects of bigelovin on tumor size.

The side effects in bigelovin and FOLFOX treatment groups were examined. H&E staining of liver, lung, heart, kidney and plasma enzyme activities of ALT, AST, LDH and CK were used to evaluate toxicity of bigelovin and FOLFOX. As shown in Fig. 6b, no apparent alterations in histological structure of lung, kidney, liver and heart tissues were observed after bigelovin and FOLFOX treatment. For the enzyme levels in plasma, bigelovin treatment did not cause obvious increases while FOLFOX significantly changed ALT and CK levels, which represented liver and muscle damage, respectively.

To further verify our *in vitro* results that bigelovin can inhibit cell proliferation and up-regulated death receptor 5, the tumors sections in control and bigelovin 20 mg/kg treatment group were stained with proliferation marker Ki 67^30^ and death receptor 5 (DR 5) antibodies using immunohistochemistry method. A significant decreased expression of Ki 67 and an increased expression of DR5 in the tumor were found after bigelovin treatment (Fig. 6c). Besides, since bigelovin was shown to inhibit angiogenesis *in vitro* in our previous study^13^, the vessels in the tumor were also stained with Factor VIII antibody^31^. Results showed that tumors in bigelovin treatment group had significantly fewer blood vessels than that in control group (Fig. 6c). TUNEL staining of tumor sections also confirmed apoptosis occurred after bigelovin treatment (Fig. 6d). In addition, DHE staining further confirmed that bigelovin treatment caused a significant increase in ROS generation in colorectal tumor tissues (Fig. 6e).

## Discussion

In the present study, we demonstrated that bigelovin, a sesquiterpene lactone, isolated from *Inula helianthus-aquatica* could induce apoptosis in colorectal cancer both *in vitro* and *in vivo*. Bigelovin had lower IC_50_ values on two colorectal cancer cells when compared to chemotherapeutic (5-Fu and cisplatin), and better selectivity for cancer cells than primary normal colon cells. Moreover, bigelovin not only inhibited proliferation, induced apoptosis, but also suppressed colony formation which indicates bigelovin treatment inhibited cell lethal- and sub-lethal damage repair. In addition, cyclin B1/CDK1 are specific G2/M phase cyclin/cdk regulatory complexes. In both cell lines, expression of CDK1 did not show apparent changes after bigelovin treatment, whereas expression of cyclin B1 was significantly increased only in HT-29 cells. Similarly, in HCT 116 cells, a trend without significant difference of increased expression of cyclin B1 could be observed in time and dose-dependent manners. Moreover, in colorectal cancer cells, bigelovin could cause G2/M arrest which was different from previous study showing that bigelovin caused G0/G1arrest in leukemia^32^ or S arrest in multiple myeloma cells^15^. The possible reason is that bigelovin is not a cell cycle specific regulatory compound but rather it down-regulated phosphorylated-retinoblastoma (p-Rb) expression in both multiple myeloma^15^ and colorectal cancer cells (which was shown in the present study). In fact, oncogenic roles of pRb/E2F1 have been well studied, and E2F1 level in multiple myeloma^15^ and colorectal cancer tumor^33^ are significant higher as compared to the corresponding non-neoplastic mucosa. Phosphorylated-Rb (p-Rb) is one of the targets of bigelovin-induced apoptosis which is consistent with previous studies.

Apoptosis occurs through two distinct and convergent pathways, one is intrinsic or mitochondrial pathway, and the other is extrinsic or cytoplasmic pathway^34^. Two pathways share a common downstream effector caspase activation and cleavage of cellular substrates such as PARP and culminates in the death. Intrinsic pathway is regulated by Bcl2 family which contains pro-apoptotic proteins (such as Bax, Bak, Bad), anti-apoptotic proteins (Bcl-2, Bcl-XL, Bcl-W)^34^, and caspase 9 is its initiator caspases. In contrast, extrinsic pathway involves activation of death receptors on the cell surface, and caspase 8 serves as initiator caspases followed by effector caspase activation. In order to address which pathway (intrinsic or extrinsic) plays crucial roles in bigelovin-induced apoptosis, expressions of a panel of proteins from both pathways were evaluated in bigelovin-treated cells. After bigelovin treatment, expression of initiator caspase activation (caspase 9) and apoptosis regulatory proteins Bax and Bcl-2, (expression of Bcl-2 in HT-29 was so low that no blot or quantitative data shown here) in intrinsic pathway changed less than proteins in extrinsic pathway (death receptors, RIP, FADD and caspase 8). Extrinsic pathway may mediate bigelovin-induced apoptosis. Among these proteins, death receptor DR5 and DcR2 dramatically up-regulated after bigelovin treatment. Although they are all death receptor, only DR5 can activate downstream caspase activation, in contrast, DcR2 (decoy receptor 2) does not mediate apoptosis induction, but can activate NF-KappaB which can be blocked by bigelovin^14^. Therefore, DcR2 may be indirect target without substantial effect and has not been selected as a considerable target for further investigation.

In our *in vitro* study, HT-29 and HCT116 cells were used mainly due to different genetic features of these two cell lines to mimic the diversity of gene mutations in CRC patients. Mutation sites in HCT 116 are TP53-wild type (wt), PTEN-wt, BRAF-wt, KRAS-mutant (G13D), PIK3CA-mutant (H1047R), while HT-29 are TP53-mutant (R273H), PTEN-wt, BRAF-mutant (V600E), KRAS-wt, PIK3CA- mutant (P449T)^35^. Among these mutations, tumor suppressor gene p53 mediates chemo-sensitivity and promotes and protects cells from oncogenesis^36^. Our study firstly demonstrated bigelovin can induce apoptosis in both p53 wt (HCT 116) and mutant (HT-29) cell lines.

In the converged data, bigelovin-induced colon cancer cell apoptosis, cell cycle arrest and DNA damage were mediated mechanistically through activation of DR5 and increase of ROS (Fig. 6f). Bigelovin increased DR5 expression and ROS to activate down-stream caspases, affected expressions of p-Rb through regulating p53, p21 and modulate cyclin B1 resulting in G2/M cell cycle arrest and cause DNA damage by upregulation of p-H2AX.

Several terpenoids share the same anti-tumor mechanisms with bigelovin such as induction of apoptosis^21^,^37^,^38^, increasing ROS^20^,^21^,^22^, up-regulation of death receptors^22^,^39^, etc. Comparing to these active terpenoids, what are the advantages of anti-tumor activity of bigelovin on colorectal cancer? From literature, our present study is a good complement to previous study on anti-tumor mechanisms of terpenoids. Firstly, although some terpenoids are reported to have anti-tumor activities, they have not been studied on colorectal cancer. For instance, plectranthoic acid, a novel and potent triterpenoid isolated from *Ficus microcarpa* with potent 5′AMP activated kinase (AMPK) activating properties induces apoptosis in prostate cancer cells^37^; tagalsins is a dolabrane-type of diterpenes that can suppresses tumor growth via ROS-mediated apoptosis on hematologic cancer cells. Secondly, some terpenoids exhibit anti-tumor effects on colorectal cancer, but activities are not better than bigelovin so they are used in combination with other drugs. For example, zerumbone has been investigated on several cancers including myeloid cancer, liver cancer, colon cancer and leukemia both *in vitro* and *in vivo*^22^,^40^. The IC_50_ value of zerumbone^41^ and bigelovin on HCT 116 cells were 30 and 0.8 μM after 48 h treatment, respectively. HT-29 was not sensitive to zerumbone treatment, whereas sensitive to bigelovin treatment. Zerumbone has been proven to treat colon cancer cells combining with TRAIL through induction of death receptors and increasing ROS. Andrographolide^42^ is another diterpenoid lactone combining with other chemotherapeutics to treat colorectal cancer due to the enhancement of cytotoxic effect. Another example is koetjapic acid, which exhibits cytotoxic effects on HCT 116 with 48 h IC_50_ value is 18.88 μg/ml (40 μM), whereas IC_50_ value of bigelovin as 0.8 μM. Finally, celastrol^20^ and triplolide^43^ are two of the most widely studied terpenoids isolated from *Thunder God Vine* with comparable or better cytotoxic effects than bigelovin. However, the toxicity^44^,^45^ including reproductive toxicity, nephrotoxicity and hepatotoxicity limited its application.

More strikingly, bigelovin significantly inhibit tumorigenesis *in vivo* and results in dose-dependent growth inhibition on tumor growth in HCT 116-tumor bearing nude mice model. This is the first demonstration that bigelovin could effectively suppress tumor growth *in vivo*. Compared to FOLFOX (first line chemotherapeutics used in cancer patients^46^), regarding to the loss of body weight in mice, bigelovin is well tolerated after ten doses of treatment although there is a little decrease in 20 mg/kg group without significance difference vs. control group. Additionally, results from H&E staining and plasma enzyme levels all revealed that there were no obvious side effects after bigelovin treatment whereas FOLFOX had severe side effects. Moreover, as expected, consistent with *in vitro* results, DR5 and ROS play a key role in bigelovin-induced apoptosis of colorectal cancer and bigelovin could inhibit proliferation and tumor angiogenesis^13^.

An ideal cancer chemotherapeutic agent should efficiently kill cancer cells without affecting normal cells. Death Reporter 5 (DR5) expression has been reported stronger in cancer cells than in normal cells^29^ indicating chemotherapeutic agent that targeted to DR5 may have less cytotoxic effect in normal cells. DR5 has been extensively validated in clinical development and it represents a new therapeutic class selectively targeting apoptosis^47^. Bigelovin exhibited anti-tumor effect though induction of DR5 suggesting its potential clinical application in colorectal cancer.

In conclusion, we showed for the first time that bigelovin induced ROS and increased DR5 expression, which in turn activated downstream caspase and caused G2/M arrest and DNA damage through regulating p21, p-Rb and p-H2AX expressions in colon cancer cells. Together with the previous findings, bigelovin exhibited anti-angiogenesis, anti-inflammation, anti-tumor activities, which lead to conclude that bigelovin could be a new multi-targeted candidate for colorectal cancer treatment.

## Materials and Methods

### Reagents and antibodies

Bigelovin (molecular weight 304.3) was prepared as previously described^13^. 5-fluorouracil (5-Fu), cisplatin, propidium iodide (PI), 3-(4,5-dimethylthiazol-2yl) 2,5-diphenyltetrazolium bromide (MTT), N-acetyl-L-cysteine (NAC) were obtained from Sigma-Aldrich (St. Louis, USA). Annexin V-FITC fluorescence kit and cell proliferation ELISA, Brdu (colorimetric) kit were purchased from Roche Diagnostics (Rotkreuz, Switzerland). All cell culture reagents were purchased from Invitrogen (Carlsbad, USA). Pan-caspase inhibitor Z-VAD-FMK was purchased from Promega (Madison, USA). Monoclonal antibodies against p53, p21, and Cyclin B1 were obtained from BD Biosciences (New Jersey, USA). Antibody against Bcl-2 was obtained from Santa Cruz Biotechnology (Dallas, USA). Antibodies of DR 5, Ki67, factor VIII for IHC were purchased from Abcam (Cambridge, UK). Other antibodies were obtained from Cell Signaling Technology, USA. *In situ* cell death detection kit, POD (TUNEL) kit and BrdU cell proliferation ELISA were obtained from Roche (Rotkreuz, Switzerland). Lysis buffer and loading buffer for Western blot were purchased from Beyotime Institute of Biotechnology (Shenzhen, China). ECL solution was purchased from GE Healthcare Life Sciences (Wauwatosa, USA). Human Death Receptor 5 siRNA (s225038 and s16756), negative control siRNA and Lipofectamin RNAiMAX Transfection Reagent and Pierce BCA protein assay kit were obtained from Thermo Scientific (Waltham, USA).

### Cell culture

Human colon adenocarcinoma cell lines HT-29 and HCT116 were obtained from ATCC (VA, USA) and cultured in McCoy’s 5A medium (ATCC) with 10% v/v Fetal bovine serum (FBS) and 1% v/v penicillin/ streptomycin (Invitrogen, USA). Primary colon cells were cultured in full-culture medium (DMEM/F12 supplemented with 30 ng/ml epidermal growth factor, 0.1 μg/ml hydrocortisone, and 10 μg/ml insulin). All cell lines were cultured at 37 °C in the presence of 5% CO_2_ and subcultured 2 to 3 times per week.

### Growth inhibition assay

MTT assays were used to determine cell viability after bigelovin treatment^48^. Briefly, cells (2500 cells/well) were seeded with culture medium in 96-well microplate and incubated at 37 °C. After 24 h incubation, cells were treated with bigelovin or positive controls for the indicated times and then 30 μL MTT (5 mg/mL) was added to each well and incubated for additional 4 h. MTT crystals were dissolved in DMSO and the absorbance was detected by a microplate spectrophotometer (μQuant, Biotek, USA). In cell proliferation assay, cells (2500 cells/well) were seeded with culture medium in 96-well microplate and incubated at 37 °C for 24 h. Cells were treated with drugs for the indicated times and the samples were analyzed as per manufacturer’s instruction. Briefly, after 24 h incubation, cells were treated with bigelovin for the indicated times and then labeled with BrdU for additional 4 h. Cells were washed, fixed, reacted with anti-Brdu and the absorbance was detected by microplate spectrophotometer (μQuant, Biotek, USA). In clonogenic assay^49^, cells were seeded at 1.5 × 10^6^ cells per 100 mm culture dish. After 24 h incubation, cells were treated at indicating doses for 48 h. Then cells were harvested, reseeded into 100 mm culture dish at a density of 1000 cells/dish and maintained for 8 days (HCT 116) or 11 days (HT-29) at 37 °C. Finally, dishes were stained with trypan blue and the colony numbers were counted. To isolate primary normal colon cells, the colon specimens collected from consented colorectal cancer patients were cut into pieces by scissors and digested in plain medium supplemented with 1 mg/ml hyaluronidase and 1 mg/ml collagenase IA-S for 15 min at 37 °C. The digestion was terminated by addition of full-culture medium and tissue suspension was dispersed and mixed well by pipetting, filtered through a 70 μm cell strainer, then transferred into a 15 ml centrifuge tube and centrifuged for 5 min at 1100 rpm. The supernatant was carefully removed and the cell pellet was suspended in full-culture medium.

### Hoechst 33258 staining for morphological changes

Cells stained Hoechst 33258 were used to evaluate morphological changes. Briefly, approximate 5 × 10^4^ cells/well were seeded in 6-well plate. After 24 h incubation, bigelovin/vehicle control was added into each well for 48 h and then cells were washed with phosphate-buffered saline (PBS). Cells were stained with 20 μg/ml Hoechst 33258 for 15 min in the dark at room temperature and morphological changes were evaluated by fluorescence microscope (Olympus IX71, Japan).

### Annexin V- PI double staining and cell cycle analysis by PI staining

An apoptosis assay was performed using the Annexin V–FITC Detection Kit (Roche, Switzerland) according to the manufacturer’s instruction by flow cytometry. For the cell cycle assay, HT-29 and HCT116 cells were cultured in 6-well plates at 2.5 × 10^5^ cells/well, and harvested after 24 and 48 h of exposure to bigelovin at indicated doses (0.7–5.4 μM). Cells were washed with PBS and ice-cold 70% v/v ethanol was used to permeabilize cell membrane at 4 °C overnight. After permeabilization, cells were resuspended in PBS containing RNase A (10 μg/ml) and PI (20 μg/ml) for 30 min in the dark at 37 °C and then analyzed by flow cytometry (10,000 events).

### Western blotting

Western blot analysis was performed, as described previously^50^. Briefly, cells (1.5 × 10^6^) were seeded in 100 mm^2^ culture dishes. After drug treatment, cells were washed with cold PBS, then gently scraping. Cells were lysed with lysis buffer and protein concentration was determined using a BCA kit. Equivalent amounts of proteins (25–35 μg) were loaded at 10% SDS-PAGE gels and transferred by blotting to polyvinylidene fluoride membranes. Membranes were blocked with 5% non-fat milk and probed with specific primary and secondary antibodies (1:1000 for primary antibodies and 1:2000 for secondary antibodies). The blots were detected using ECL solution and were captured by a molecular imager, ChemiDoc XRS+ (Bio-Rad, USA). The bands intensities were quantified using ImageJ (NIH, USA). The intensities of bands were normalized to its own internal standard proteins (beta-actin) for each protein samples. The quantitative data presented as fold of untreated control.

### SiRNA transfection

For siRNA transfection, HT-29 and HCT 116 cells were plated in 6-well plates/60 mm dish and cultured for 16 h. Thirty pmol siRNAs (s225038 and s16756) /negative control, 9 μL Lipofectamine™ RNAiMAX were mixed with 150 μL Opti-MEM medium separately for 5 min. Lipofectamine was added to siRNAs for 20 min of incubation. Culture medium of HT-29 and HCT 116 cells was changed to Opti-MEM medium and then a mixture of siRNAs were added to cells. After 24 h of incubation, culture medium was changed to fresh, full-culture medium plus bigelovin/ vehicle DMSO at the indicated dose for 24 h.

### *In vivo* xenograft studies

All animal experiments were carried out under institutional guidelines, and experimental procedures were approved by the Animal Experimentation Ethics Committee of The Chinese University of Hong Kong (Ref. No. 14/170/MIS). Male BALB/c nude mice (6–8 weeks of age) were supplied by the Laboratory Animal Services Centre of CUHK. Mice were bred and maintained in pathogen-free conditions (sterile water and food) in specifically designed air-controlled rooms with a 12-h light/dark cycle. HCT 116 cells (5 × 10^6^) were suspended in 100 μL PBS and injected subcutaneously into the back of nude mice. After tumor reaching 50 mm^3^ in volume, mice were randomized into groups of 5 animals and treated with either vehicle, bigelovin (5 mg/kg, 10 mg/kg, and 20 mg/kg) or FOLFOX (15 mg/kg fluorouracil daily; 5 mg/kg folinic acid daily and 5 mg/kg oxaliplatin once a week) as positive control. Bigelovin or vehicle was administered intraperitoneally every two days for 10 times while FOLFOX was administered intraperitoneally daily for 7 days. Tumors were measured twice per week using calipers and tumor volumes were calculated using the formula length × width × depth/2 (mm^3^), and at the same time, each mouse was weighed. Bigelovin was dissolved in PBS (with 2% DMSO). At the end of experiment, plasma, tumors, livers, hearts, kidneys and lungs were collected. The plasma enzymes, aspartate aminotransferase (AST), alanine aminotransferase (ALT) and lactate dehydrogenase (LDH) were used to assess tissue damage; creatine kinase (CK) was used to assess muscle damage. They were evaluated according to manufacturer’s instructions (Stanbio Laboratory, USA).

### Immunohistochemistry

Tumor tissue specimens were collected from mice and separated into two halves, one half was fixed in 10% formalin and embedded in paraffin whereas the other half was embedded in Optimal Cutting Temperature compound (OCT) and frozen at −80 °C. Other organs (liver, lung, heart, kidney) were fixed in 10% formalin, embedded in paraffin and sectioned at 5 μm, collected on coated slides for staining. Tumor sections on slides were dewaxed, rehydrated, incubated with Cova Decloater and incubated with primary antibodies (Ki 67, DR5, Factor VIII) overnight at 4 °C as described in other study^51^. Formalin-fixed paraffin embedded sections were also used in TUNEL assay as per kit’s instruction. Frozen sections of tumors were sectioned at 10 μm using a cryostat (CM 1100, Leica, Germany), and stained with dihydroethidium (DHE, 2.5 μM in Krebs-HEPES buffer) dye for 15 min at 37 °C in the dark^50^. Fluorescence captured by fluorescence microscope (Olympus IX71, Japan) and intensity of fluorescence was calculated by Image J software (Image J 1.50b).

### Statistical analysis

All *in vitro* experiments were performed at least three times. Quantitative results were analyzed by Student’s *t* test or one-way ANOVA. Statistical significance was considered when *p* < 0.05. All statistical analysis was assessed by SPSS 20 software.

## Additional Information

**How to cite this article:** Li, M. *et al*. Bigelovin triggered apoptosis in colorectal cancer *in vitro* and *in vivo* via upregulating death receptor 5 and reactive oxidative species. *Sci. Rep.* **7**, 42176; doi: 10.1038/srep42176 (2017).

**Publisher's note:** Springer Nature remains neutral with regard to jurisdictional claims in published maps and institutional affiliations.

## Supplementary Material

Supplementary Information
