# Supplementary material for: Bigelovin triggered apoptosis in colorectal cancer in vitro and in vivo via upregulating death receptor 5 and reactive oxidative species
Source: Sci Rep. 2017 Feb 9;7:42176. doi: 10.1038/srep42176 (PMC5299840; doi:10.1038/srep42176)

Bigelovin triggered apoptosis in colorectal cancer *in vitro* and *in vivo* via upregulating death receptor 5 and reactive oxidative species

Mingyue Li[1,2,#], Li-Hua Song[5,#], Grace Gar-Lee Yue[2,3], Julia Kin-Ming Lee[2,3], Li-Mei Zhao[6], Lin Li[4], Xunian Zhou[1,2], Stephen Kwok-Wing Tsui[1], Simon Siu-Man Ng[4], Kwok-Pui Fung[1,2,3], Ning-Hua Tan[5,6,]*, Clara Bik-San Lau[2,3,]*

[1]School of Biomedical Sciences; [2]Institute of Chinese Medicine; [3]State Key Laboratory of Phytochemistry and Plant Resources in West China (CUHK); [4]Department of Surgery, The Chinese University of Hong Kong, Shatin, New Territories, Hong Kong; [5]School of Traditional Chinese Medicines, China Pharmaceutical University, Nanjing 211198, China; [6]State Key Laboratory of Phytochemistry and Plant Resources in West China, Kunming Institute of Botany, Chinese Academy of Sciences, Kunming 650201, China.

*Corresponding authors. Email: claralau@cuhk.edu.hk; nhtan@mail.kib.ac.cn

Supplementary Data

Supplementary Figure S1. Bigelovin induced p21 and p53 expression. A and B, cells were treated with bigelovin (5.4 μM for HT-29 and 4.2 μM for HCT 116 cells) for indicated time. Whole-cell extracts were prepared and analyzed by Western blot using antibodies against p21 and p53. Right panel in (A) and (B) are quantitative data. The data was quantified and represented in mean ± SD for independent 3 experiments; *,P<0.05, **, P<0.01, ***, P<0.001 vs. control at the corresponding time point.

Table S1. IC_{50} values on cancer and colon normal cells

	Bigelovin (μM)			5-Fu (μM)			Cisplatin (μM)		
	24 h	48 h	72 h	24 h	48 h	72 h	24 h	48 h	72 h
HT29	1.83±0.47	1.21±0.17	1.08±0.16	> 27	15.83±10.6	7.63±3.8	> 27	> 27	> 27
HCT116	1.38±0.29	0.8±0.17	0.77±0.25	> 27	4.78±1.67	1.69±0.22	> 27	> 27	> 27
Normal primary colon cells		8.55±1.84							

Supplementary figure S1

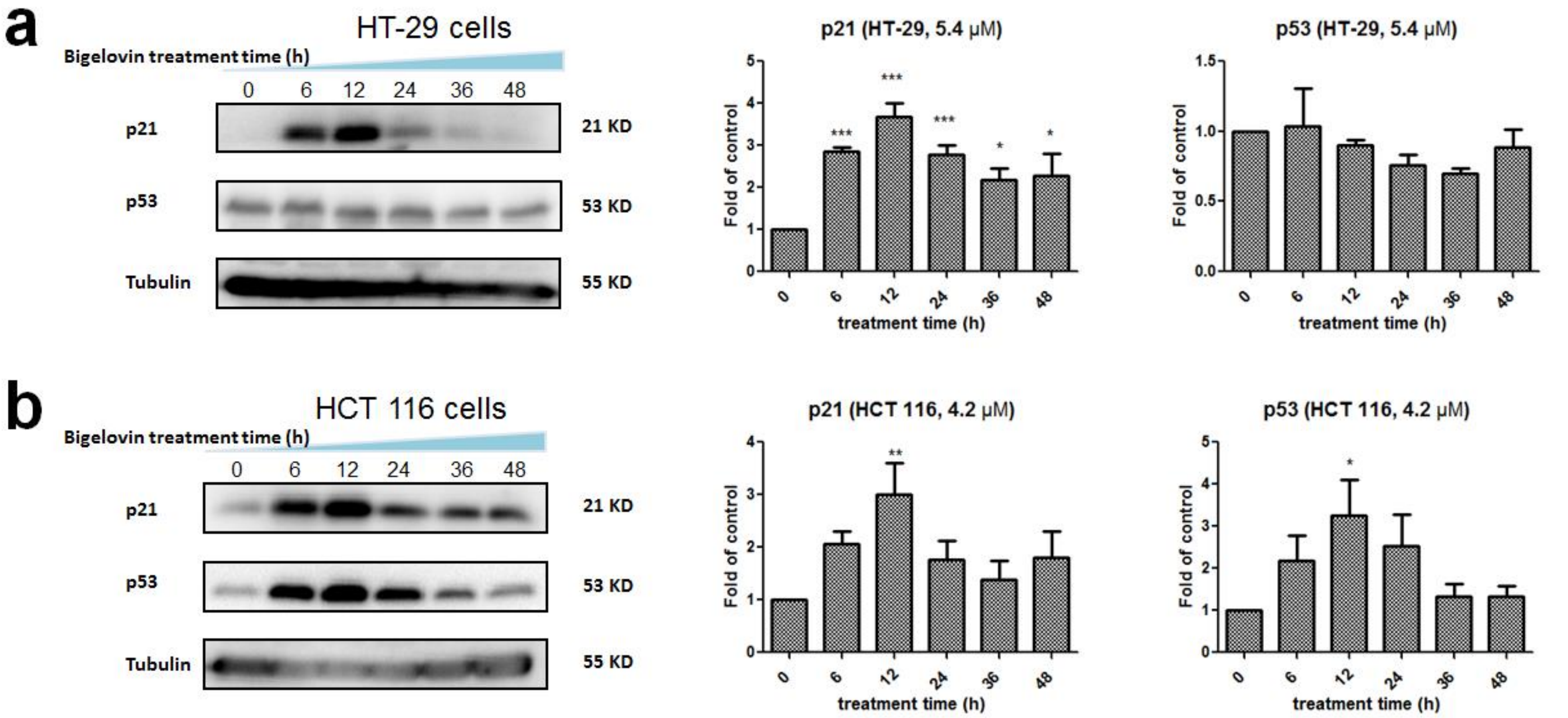

Supplement: Supplementary Information [file srep42176-s1.pdf]
